# Prognosis of cholangiocarcinoma patients based on multiple patterns of programmed cell death, integrated analysis of the immune microenvironment and drug sensitivity

**DOI:** 10.3389/fgene.2025.1457204

**Published:** 2025-05-14

**Authors:** Yupeng Xu, Jian Sun, Xin Yan, Zhenliao Mao, Yiming Chen

**Affiliations:** ^1^ Department of Surgery, The Second Affiliated Hospital and Yuying Children’s Hospital of Wenzhou Medical University, Wenzhou, Zhejiang, China; ^2^ The First Clinical Medical College, Wenzhou Medical University, Wenzhou, China

**Keywords:** cholangiocarcinoma, programmed cell death, prognostic model, tumor microenvironment, RNA-seq

## Abstract

**Background:**

Cholangiocarcinoma (CHOL) is a highly malignant bile duct cancer with a poor prognosis and rising incidence. Programmed cell death (PCD) plays a crucial role in cancer biology, influencing tumor immunity and treatment response. This study analyzes the impact of multiple PCD patterns on CHOL prognosis, tumor microenvironment (TME) and drug sensitivity.

**Methods:**

RNA sequencing data from TCGA-CHOL and GSE107943 were analyzed to identify PCD-related genes. A PCD-associated Risk Score was constructed using Cox and Lasso regression analyses. The score’s prognostic value was assessed through survival analysis, ROC curves, and functional annotation.

**Results:**

We identified 111 differentially expressed PCD-related genes, including *NCK2*, *BNIP3* and *BIK*, that constituted PCD-associated Risk Score and correlated with prognosis of CHOL. Functional analyses indicated enrichment in immune-related processes. High-risk patients showed increased immune cell infiltration and higher immune checkpoint expression, suggesting a benefit from immunotherapy. They also demonstrated greater sensitivity to several chemotherapeutic and targeted agents.

**Conclusion:**

PCD-associated Risk Score is a robust prognostic tool for CHOL, influencing TME modulation and therapeutic response, and may guide personalized treatment strategies.

## 1 Introduction

Cholangiocarcinoma (CHOL) is a rare highly malignant neoplasm originating from the epithelial cells of the bile ducts (Razumilava and Gores, 2014). According to statistics, although cholangiocarcinoma accounts for approximately 3% of all gastrointestinal cancers, its incidence has been rising globally (Khan et al., 2019; Patel, 2002). Therapeutic approaches for cholangiocarcinoma are largely dictated by the tumor’s location and stage at diagnosis (Blechacz et al., 2011; Nakanuma et al., 2023). Surgical resection remains the only possible cure but is feasible in a minority of patients due to the terminal stage at presentation (Ilyas et al., 2018; Roskoski, 2023). Despite advances in drug therapy, the overall prognosis for CHOL remains poor, with a 5-year survival rate of about 5 percent for patients with terminal disease (Roskoski, 2023; Kelley et al., 2020; Greten et al., 2023). Therefore, there is an urgent need to investigate further biomarkers that could potentially improve early diagnostic accuracy and drug therapy efficacy.

Programmed cell death (PCD) is a common process in living organisms. It is involved in a variety of biological events, including immunity, maintenance of tissue homeostasis, and elimination of harmful cells (Sun and Peng, 2009; Gong et al., 2023). PCD consists of multiple mechanisms of complex and interdependent cell death, including apoptosis, ferroptosis, necroptosis, pyroptosis, entotic cell death, netotic cell death, lysosome-dependent cell death, parthanatos, autophagy, oxeiptosis, cuproptosis, alkaliptosis, and disulfidptosis (Bedoui et al., 2020; Zheng et al., 2023; Zou et al., 2022). Among these PCDs, ferroptosis and autophagy, have been the hot spots of research in recent years. Redox imbalance of oxidants and antioxidants due to lipid peroxidation is an important cause of ferroptosis, which can overcome the infinite replication and immortalization of tumor cells to some extent (Zhao et al., 2022; Tang et al., 2021). Autophagy is the main mechanism that mediates the delivery of various cellular cargoes to lysosomes for degradation and recycling, And different degrees of cellular autophagy play a promoting or inhibiting role in tumors (Miller and Thorburn, 2021; Debnath et al., 2023). Thus by achieving precise regulation of programmed cell death may be a promising target for cancer therapy. In addition, since disorders of PCD in tumors lead to dysregulation of the number or function of immune cells in the tumor microenvironment thereby leading to immunogenic or non-immunogenic responses, which ultimately lead to tumor regression or progression (Liu J. et al., 2022; Ke et al., 2016; Pitt et al., 2017). Many studies over the past few years have revealed the mechanism of action of a specific PCD pattern in cholangiocarcinoma (Wang et al., 2022; Sarcognato et al., 2020; D’Artista and Seehawer, 2025). In addition, one study utilized extensive sequencing data to investigate the characteristics associated with ferroptosis in patients with cholangiocarcinoma and developed an accurate prognostic risk model (Yao et al., 2022). However, given the complex crosstalk between PCDs, the specific role of the PCD patterns in CHOL has not been fully elucidated. Therefore, elucidating how PCD is regulated in cholangiocarcinoma and the complex molecular mechanisms involved in tumor immunity and exploring the role of these mechanisms in cancer progression are essential to identify innovative therapeutic targets and strategies for cholangiocarcinoma.

This study used bioinformatics techniques to identify PCD-related genes in cholangiocarcinoma by integrating multiple expression profiling data. With these genes, we established a new predictive signature called PCD-associated Risk Score, which can better identify prognostic features and alterations in the immune microenvironment of cholangiocarcinoma patients. In conclusion, this paper dissects the key genes and different subtypes affecting the prognosis of CHOL from a PCD perspective, providing valuable guidance for optimal treatment options.

## 2 Methods

### 2.1 Data collection and acqusition of PCD-related gene

Since large-scale genomics projects are generally stored in The Cancer Genome Atlas (TCGA, https://portal.gdc.cancer.gov/) and Gene Expression Omnibus (GEO, https://www.ncbi.nlm.nih.gov/geo/), we collected RNA sequencing (RNA-seq) data and corresponding clinical and prognostic information for a total of 74 specimens from TCGA-CHOL and GSE107943 (https://www.ncbi.nlm.nih.gov/geo/query/acc.cgi?acc=GSE107943), which finally contained 65 tumor samples and 9 normal samples. For data from both databases, we finally converted to TPM to calculate gene expression and removed batch effects between the two sets of data by using the combat algorithm. Differential expression analyses were performed using the “limma” R package with thresholds of adjusted p-values <0.05, |log2FC| > 2. Then, we obtained experimentally validated PCD-related genes from the past published literature (Qin et al., 2023).

### 2.2 Construction of PCD-associated risk score

To identify the key PCD genes affecting prognosis, all cholangiocarcinoma patients were equally randomized into a test set and a validation set. We filtered for genes associated with prognosis using a one-way Cox in the training set (p < 0.05), then performed Lasso regression analyses on these genes, and finally, to determine the optimal prognostic PCD signature, stepwise Cox regression was used to calculate the regression coefficients of the genes to optimize the model.

$$\text{Risk score}=\sum_{1}^{n}coef_{i}\;*\;x_{i}.$$

x_i_ and coef_i_ denote the expression of each PCD-related genes and its corresponding coefficient, respectively. This formula is applied to calculate the staging score for each patient with suspicious cell carcinoma and to distinguish high-risk patients from low risk patients based on the median of PCD-associated Risk Score.

### 2.3 Construction of the nomogram based on the predictive signature

We constructed a nomogram comprising risk scores and clinical characteristics such as age, gender and stage. This nomogram generates a risk score for each patient and enhances the accuracy of prognostic predictions.

### 2.4 Evaluation of the PCD-associated risk score

Survival analysis was conducted using the “survival” R package, based on high and low-risk groups derived from nomogram scores. Subsequently, Kaplan-Meier survival curves were plotted with the “survminer” R package to compare patient outcome distributions between the two groups. Additionally, the risk model’s effectiveness and efficiency were validated through 1-, 2-, and 3-year receiver operating characteristic (ROC) curve analyses using the “ROC” R package.

### 2.5 Functional annotation and pathway enrichment analysis

We utilized the “limma” R package to analyze the differentially expressed genes in patients with high and low risk scores, with a cutoff of |log2 (fold change)| >1 and p-value <0.05. In addition, the Gene Ontology (GO) and the Kyoto Encyclopedia of Genes and Genomes (KEGG) analyses were applied to determine the roles of these genes with the “clusterProfiler” R package (adjusted p-value <0.05).

### 2.6 Immune infiltration analysis

In order to understand the immune infiltration in tissues, five advanced algorithms (CIBERSORT, QUANTISEQ, XCELL, TIMER and EPIC) were applied to estimate the relative abundance of tumor-infiltrating immune cells. Meanwhile, we used ESTIMATE to calculate StromalScore, ImmuneScore, and ESTIMATEScore for tumor microenvironment analysis.

### 2.7 Analyses of somatic mutations

We extracted the somatic mutation data of CHOL patients from the TCGA database and then used the “maftool” R package to calculate the mutation frequency of each sample and visualize the somatic mutations, and finally mapped the OncoPlot of the top 10 genes.

### 2.8 Correlations of predictive signature and drug sensitivity

To assess the potential of this predictive signature for CHOL therapy, we used GDSC data to predict the response of CHOL patients to drugs commonly used for clinical treatment. We used the “oncoPredict” R package to determine the half-maximal inhibitory concentration (IC50) of each drug. The Wilcoxon test was then applied to compare the drug treatment sensitivity between the two groups.

### 2.9 Single-cell sequencing analysis of PCD-related genes

The GSE138709 used in the article was obtained from the GEO database, which contains five cholangiocarcinoma tissues and three adjacent tissues. The “Seurat” R package was used for the initial processing of the data, filtering the cells according to mitochondrial gene expression of less than 5 percent, and expression of genes greater than 200 and less than 5,000. The top 2,000 highly variable genes were extracted using the “FindVariableFeatures” function, then the cells were clustered using the “RunPCA” function for dimensionality reduction, and finally we annotated the cell type based on the gene expression characteristics of each cluster.

## 3 Results

### 3.1 Identification of PCD-related genes in patients

**FIGURE 1:**
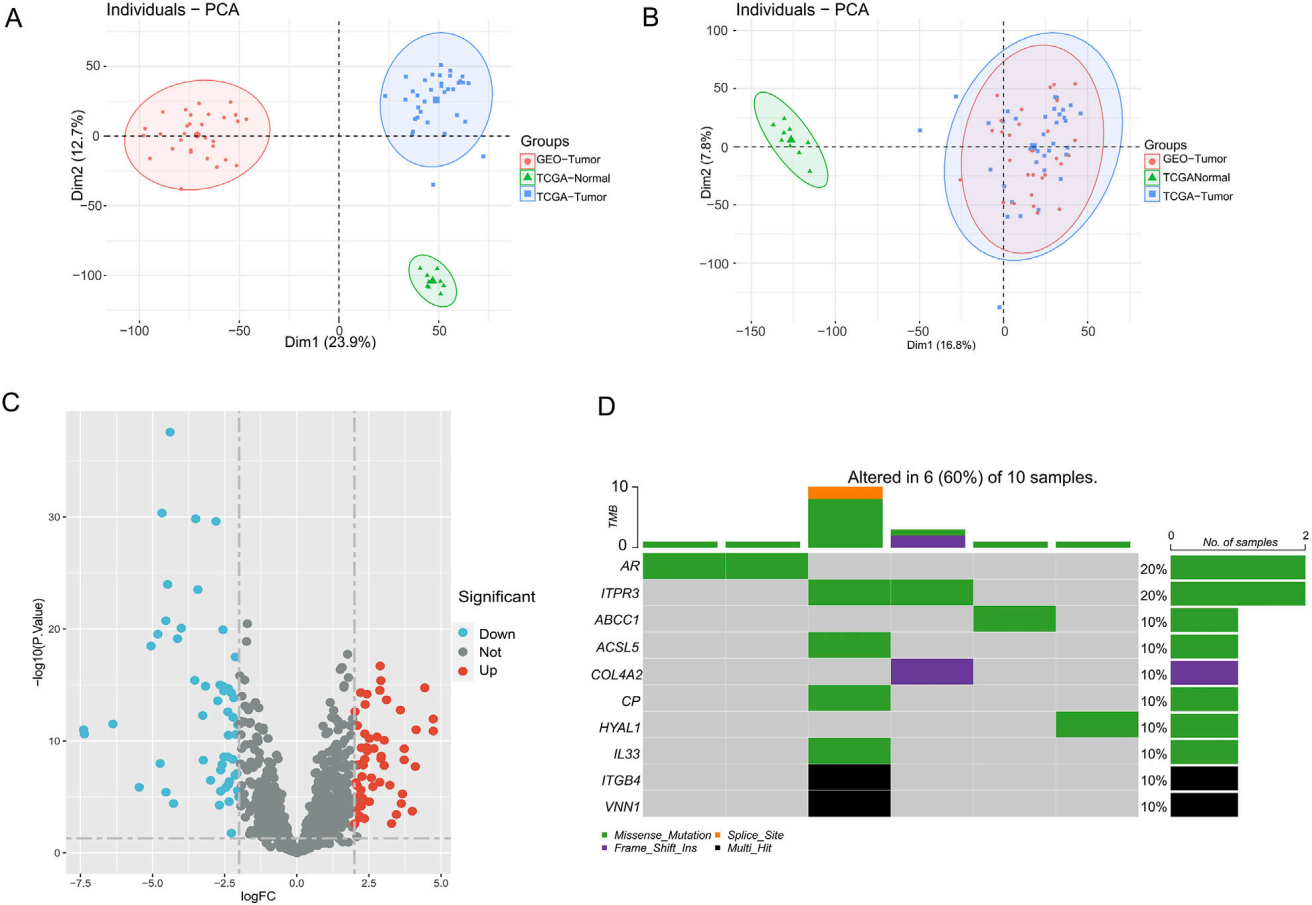
PCD-related genes landscape in CHOL patients. **(A,B)** PCA plots before and after combat batching. **(C)** Volcano map of PCD-related genes differentially expressed in cholangiocarcinoma. **(D)** Somatic mutation map of PCD-DEGs in CHOL patients.

With the “COMBAT” algorithm, we eliminated the batch effect between samples and merged the two sets of cohort data (Figures 1A,B). We obtained a collated 1964 genes related to the 18 PCD patterns from previous studies, followed by differential analyses in the TCGA-CHOL and GSE cohorts ultimately yielding 111 differentially expressed genes (DEGs, |log2FC| > 2, adjust p-values <0.05). These genes were derived from 13 different PCD patterns (Figure 1C; Supplementary Table S1). Heatmap presents scaled expression levels of 111 DEGs (Supplementary Figure S1A), while the protein-protein interaction network of the DEGs is depicted in Supplementary Figure S1B, the redder the color of a protein, the more strongly it is associated with other proteins. In addition, we delved into mutations of PCD-associated genes in the TCGA cohort of CHOL patients, with AR and ITPR3 having the highest mutation grade (20%). (Figure 1D).

### 3.2 Construction of PCD-associated risk score

**FIGURE 2:**
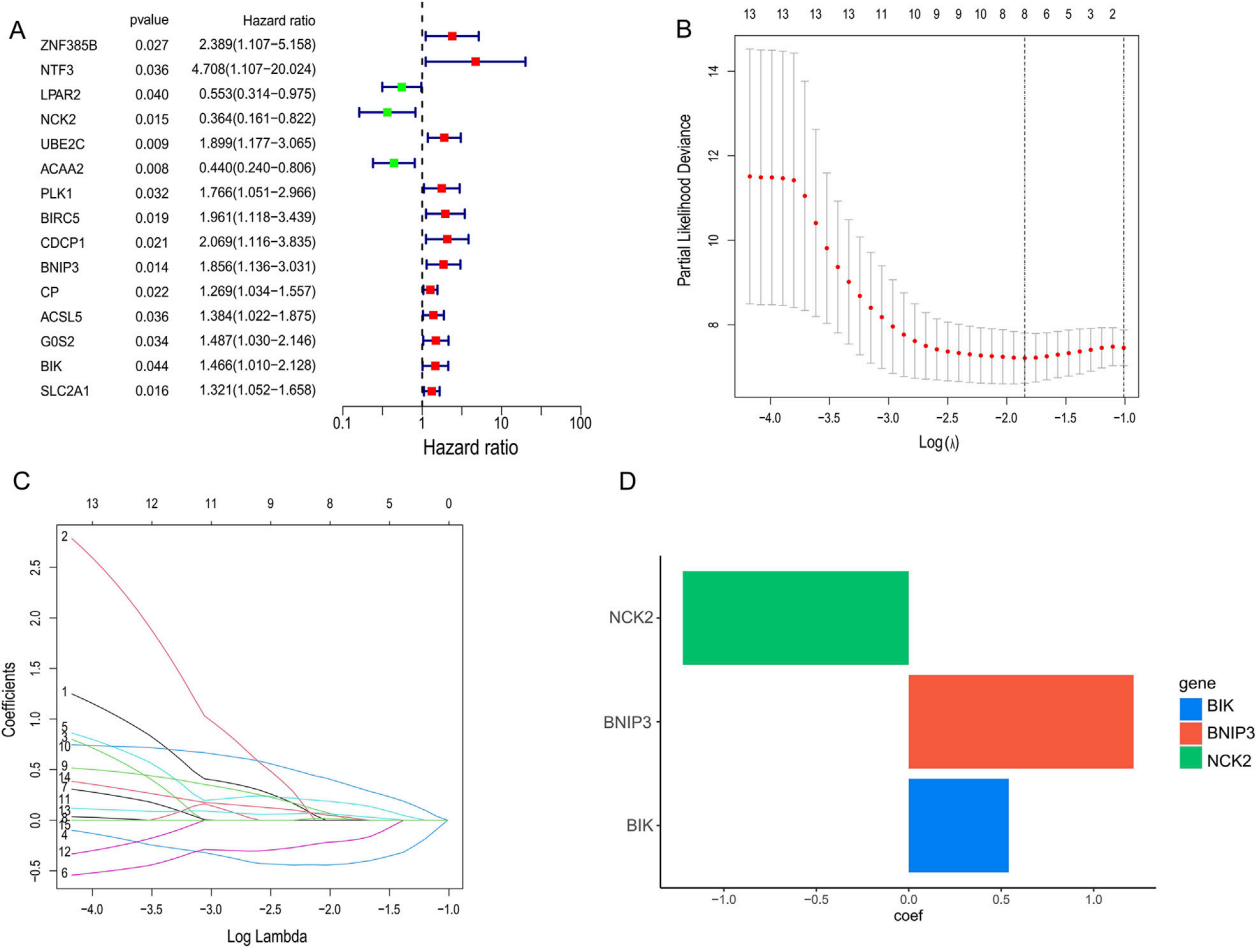
Identification of candidate genes for PCD-associated Risk Scores. **(A)** COX regression screening for genes associated with prognosis. **(B,C)** Selection of optimal parameters (lambda) to construct the LASSO model. **(D)** Stepwise cox regression to determine the coefficient of gene.

Tumour samples were randomly divided into a validation set and a training set, in which 15 PCD genes associated with prognosis were identified using one-way Cox regression. Among these 15 genes, only *NCK2*, *LPAR2* and *ACAA2* are protective factors and the rest are risk factors (Figure 2A). Subsequently, to prevent over-fitting, we used Lasso regression and acquired the 3 hub PCD-related genes (*NCK2*, *BNIP3*, *BIK*) based on the best lambda values (Figures 2B,C). Ultimately, we used stepwise Cox regression to determine the regression coefficients for each gene and constructed a PCD-associated Risk Score. The formula for the PCD-associated Risk Score presents the regression coefficients for each gene (Figure 2D).

### 3.3 PCD-associated risk score is associated with prognosis in CHOL patients

**FIGURE 3:**
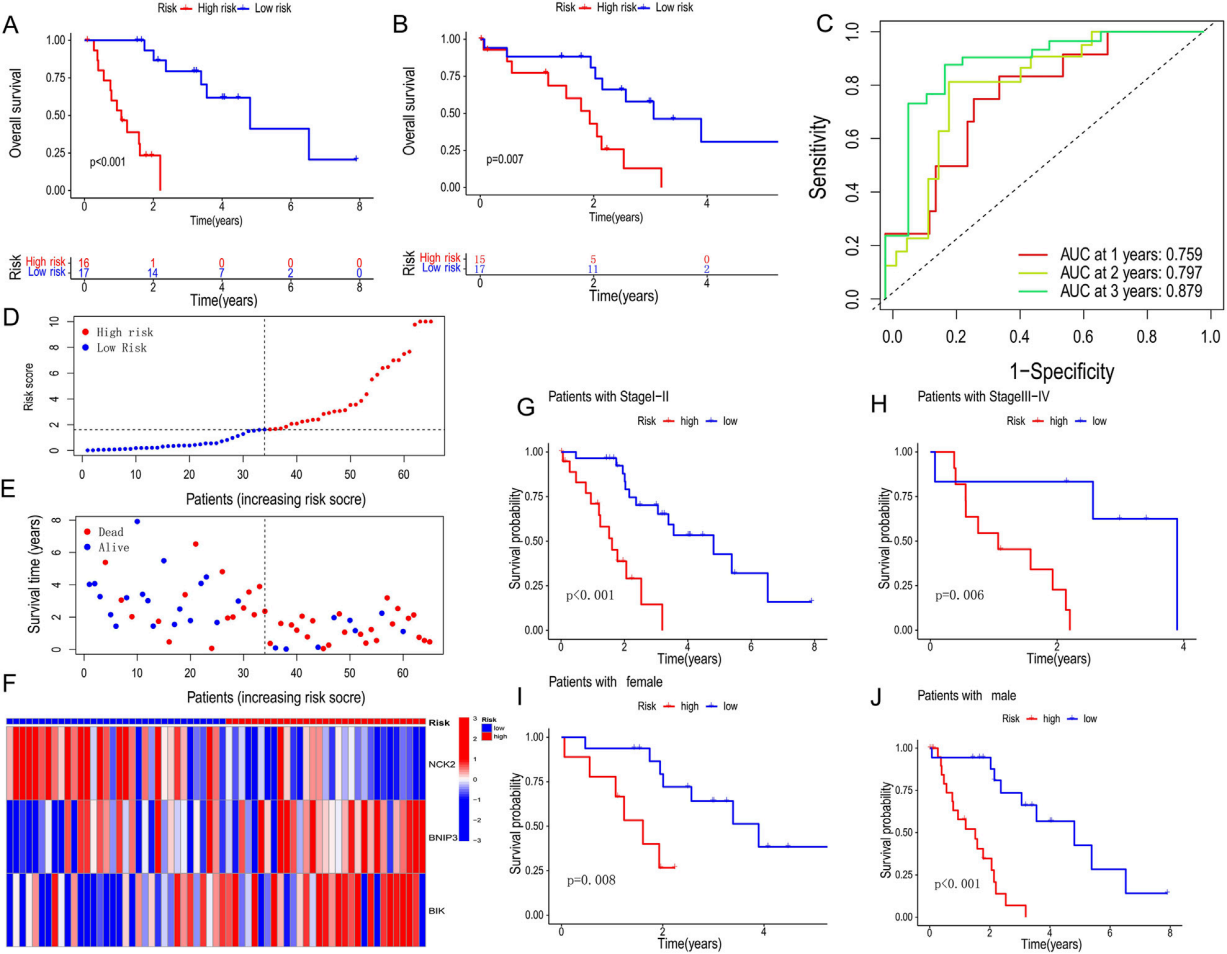
Prognostic characteristics between high and low PCD-associated Risk Score groups. **(A,B)** Survival curves for training and validation sets for high and low PCD-associated Risk Score groups. **(C)** Receiver operating characteristic (ROC) analysis of 1-, 2- and 3-year survival in all CHOL patients. **(D,E)** PCD-associated Risk Scores according to survival status and time distribution. **(F)** Heatmap of hub PCD genes expression in high and low PCD-associated Risk Score groups. **(G–J)** Survival curves for different pathological and gender subgroups.

Using the PCD-associated risk scores calculated from the training set, we categorized patients into high- and low-risk groups based on the median of the risk scores. The results revealed a shorter survival time and worse prognosis in the high-risk group in both the validation and training sets (p < 0.001, p = 0.007) (Figures 3A,B). Across the cohort, we observed a reduction in survival samples and an increase in death samples when risk scores were elevated (Figures 3D,E). The genes involved in constructing the score, *BNIP3*, and *BIK*, were highly expressed in the high-risk group in contrast with *NCK2* (Figure 3F). Meanwhile, the accuracy and reliability of the PCD SCORE in prognosis prediction were proved by the ROC curves (AUC = 0.759, 0.797, 0.879 for 1-, 2-, and 3-year) (Figure 3C). To assess the stabilization of the PCD-associated Risk Score, we reassessed its predictive prognostic ability across gender and pathological stage, and showed that the signature continues to perform excellently (Figures 3G–J).

### 3.4 Development and evaluation of the nomogram survival model

**FIGURE 4:**
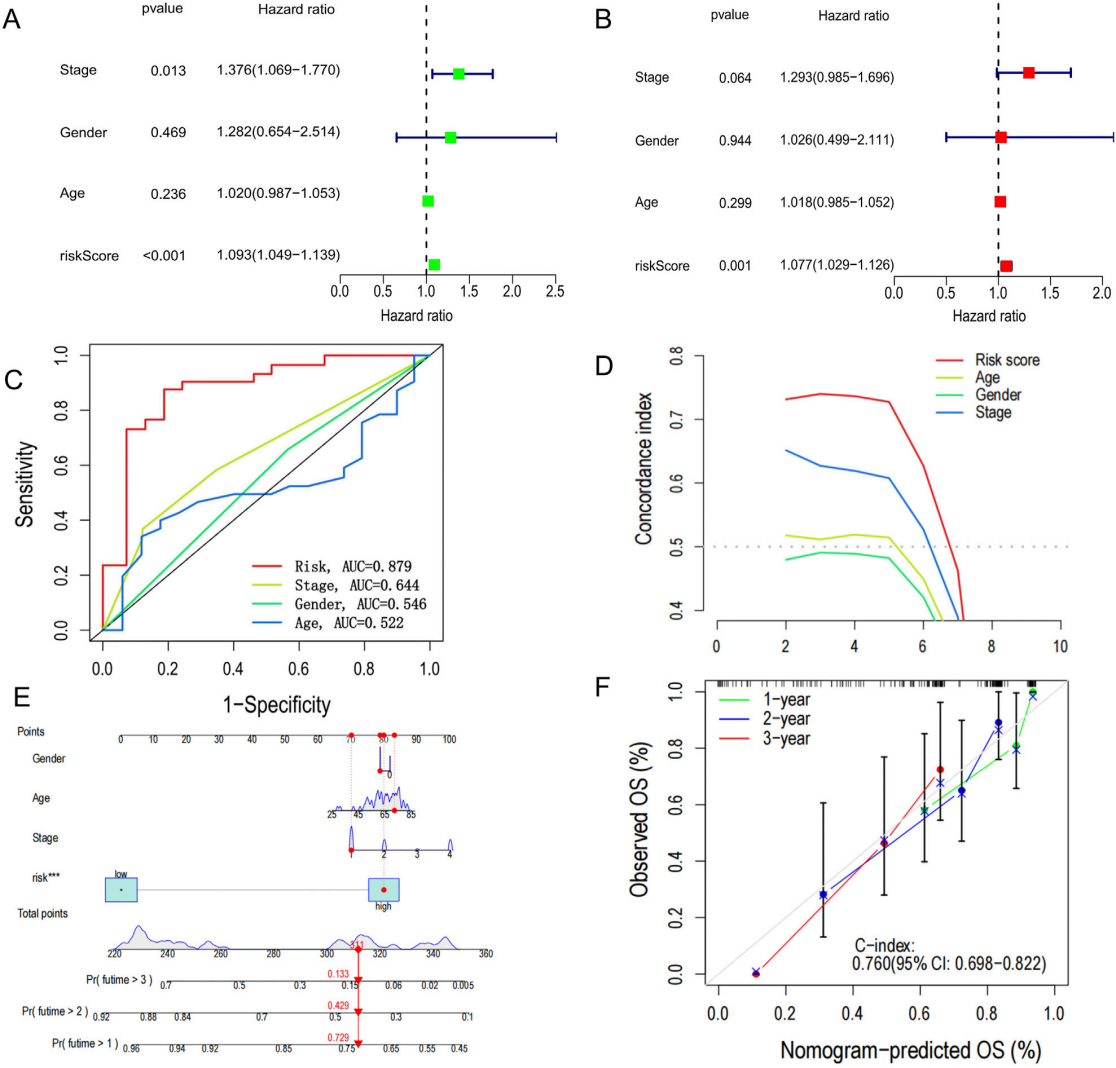
Construction of nomogram survival model. **(A)** Univariate analysis of clinicopathological features and PCD scores in all CHOL patients. **(B)** Multivariate analysis of clinicopathological features and PCD scores in all CHOL patients. **(C)** Receiver operating characteristic (ROC) analysis of PCD-related risk scores for all CHOL patients. **(D)** C-index versus time plot. **(E)** Establishment of a nomogram survival model chart to predict prognosis in patients with CHOL. **(F)** C-index curves at 1-, 2- and 3- years for nomogram survival model for all CHOL patients.

We performed both univariate and multivariate Cox regression analyses to determine whether PCD-associated Risk Score could serve as an independent prognostic factor. The results showed that PCD-associated Risk Score was a significant risk factor in one-way Cox regression (HR = 1.093, 95% CI: 1.049–1.139, P < 0.001, Figure 4A). Besides, PCD-associated Risk Score was still suggested to have an independent prognostic value after a multifactorial Cox analysis adjusting for other confounding factors (HR = 1.077, 95% CI: 1.029–1.126, P = 0.001) (Figure 4B). The area under the curve of the ROC for PCD-associated Risk Score suggests that PCD-associated Risk Score has better predictive power compared to other clinical features (Figure 4C). These results were also confirmed by the c-index (Figure 4D). To further enhance the predictive power of the signature, we incorporated several clinical features to create a nomogram to predict 1-, 2-, and 3-year patient survival rates (Figure 4E). The ability of the nomogram survival model to accurately predict 1-, 2- and 3-year survival is proven by calibration curves (Figure 4F).

### 3.5 Differences in functional and clinical characteristics across risk groups

**FIGURE 5:**
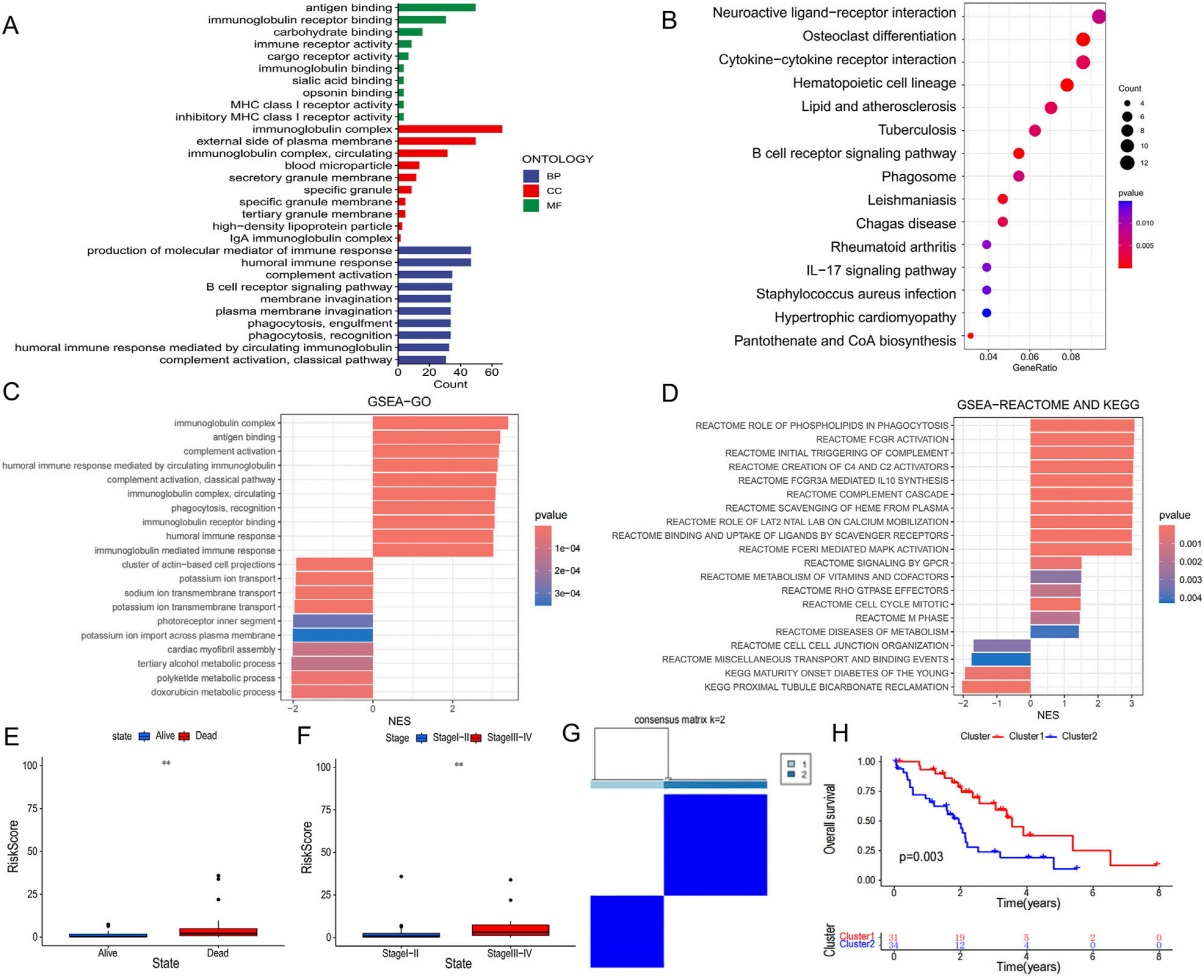
Differences in biological function and clinical characteristics of high and low PCD-associated Risk Score groups. **(A)** Results of GO analysis of differentially expressed genes between high and low PCD-associated Risk Score groups. **(B)** Results of KEGG analysis of differentially expressed genes in high and low PCD-associated Risk Score groups. **(C,D)** GSEA analysis between high and low PCD-associated Risk Score groups. **(E,F)** Differences in PCD-associated Risk Scores between patients with different clinical characteristics. **(G)** All CHOL patients were classified into two molecular clusters based on hub PCD genes. **(H)** Differences in survival curves in patients with two molecular clusters.

We used the “limma” R package to identify differentially expressed genes (DEGs) between high- and low-risk groups based on the criteria of |log2FC| > 1 and p < 0.05. To explore the biological reasons for the prognostic differences between the high and low risk groups, we used GO and KEGG analyses. GO analysis showed DEGs are enriched in immune-related biological processes such as antigen binding, immunoglobulin complex and production of molecular mediators of the immune response, which are closely related to tumor immunity (Figure 5A). Interestingly, KEGG analysis results were also enriched for some immune-related signaling pathways such as cytokine-cytokine receptor interaction and B cell receptor signaling pathway (Figure 5B). In addition, we performed GSEA analyses and presented the 10 results for the largest and smallest NES (Figures 5C,D). In terms of clinical characteristics, PCD-associated Risk Score with significant differences in different outcomes and pathological stages suggested a strong association between them (Figures 5E,F). Additionally based on the signature genes, we identified two clusters by unsupervised clustering, with Cluster one having a better prognosis (Figures 5G,H).

### 3.6 ScRNA-seq analysis

**FIGURE 6:**
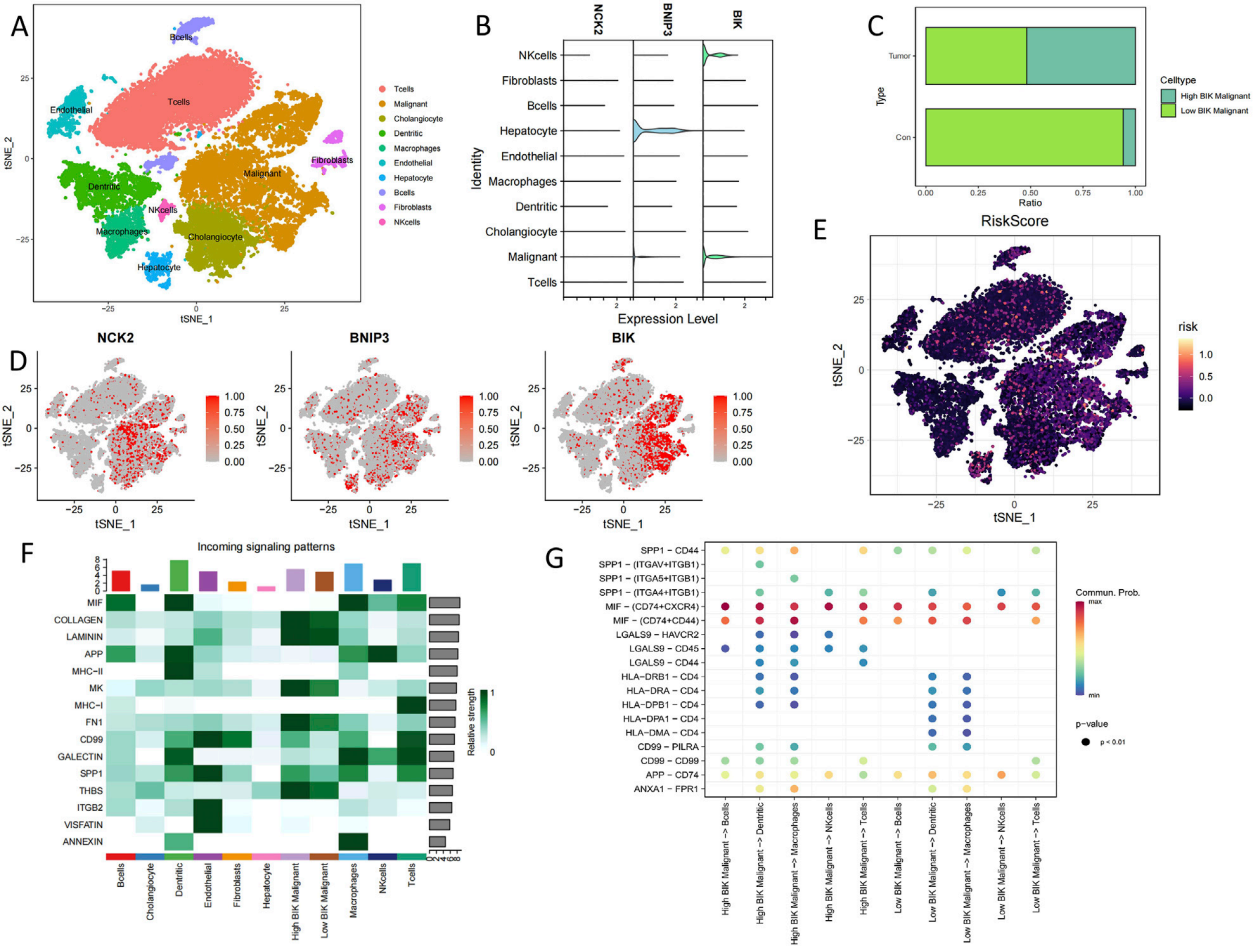
Single-cell sequencing analysis of cholangiocarcinoma patients. **(A)** Annotated tsne plot of cell types. **(B)** Violin plots of hub PCD genes expression in various cells type. **(C)** Proportion of specific cells in tumour and normal samples. **(D)** Tsne plots of hub PCD genes expression in various cells type. **(E)** Tsne plots of PCD-associated Risk Scores in various cells type. **(F)** Heatmap of the contribution of incoming signalling patterns received by various cell type. **(G)** Bubble plot of ligand-receptor strength between cells.

All cells from 5 cholangiocarcinoma tissues and 3 from para carcinoma tissues were identified as 10 lineage cells (T cell, malignant, cholangiocyte, dendritic, macrophage, endothelial, hepatocyte, B cell, fibroblast, NK cell) (Figure 6A). Violin plot and tsne plot illustrate the expression of the 3 model genes in cells (Figures 6B,D). In addition, the tsne plot reveals a higher PCD-associated Risk Score for Tcells and Maligant (Figure 6E). Since *BIK* is highly expressed in maligant, we classified maligant into high *BIK* maligant and low *BIK* maligant based on the median *BIK* expression in maligant, The results showed a substantial increase in the proportion of high *BIK* maligant in tumor samples (Figure 6C). To observe the relationship between *BIK* and immune cells we performed cellchat analysis, the results showed that *MIF*, *APP*, *SPP*, *MHC-Ⅰ*, *GALECTIN* and *CD99* signaling contributed more in immune cells, in which eventually found that *LGAS9-HAVCR2*, *CD99-CD99*, and *SPP1*-*CD44* ligand-receptor pairs were significantly active between high *BIK* malignant and immune cells (Figures 6F,G).

### 3.7 Differences in immune characteristics between high and low risk groups

**FIGURE 7:**
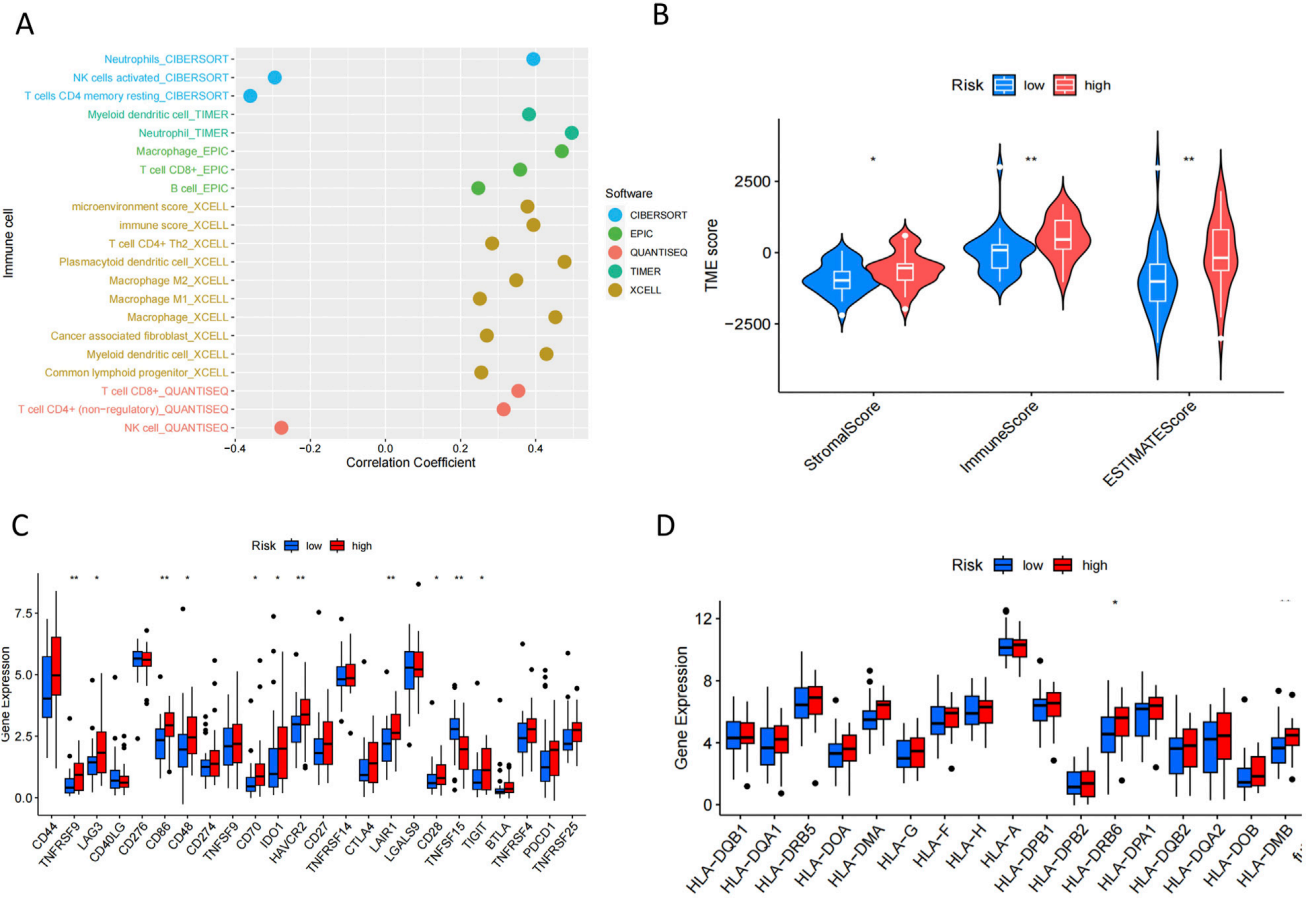
Immunological characterisation in patients with CHOL. **(A)** Correlation plots of immune cell abundance and PCD-associated Risk Scores predicted by five algorithms. **(B)** Differences in TME Score between groups. **(C,D)** Differences in the expression of immune checkpoint genes and HLA-associated genes between different groups.

**FIGURE 8:**
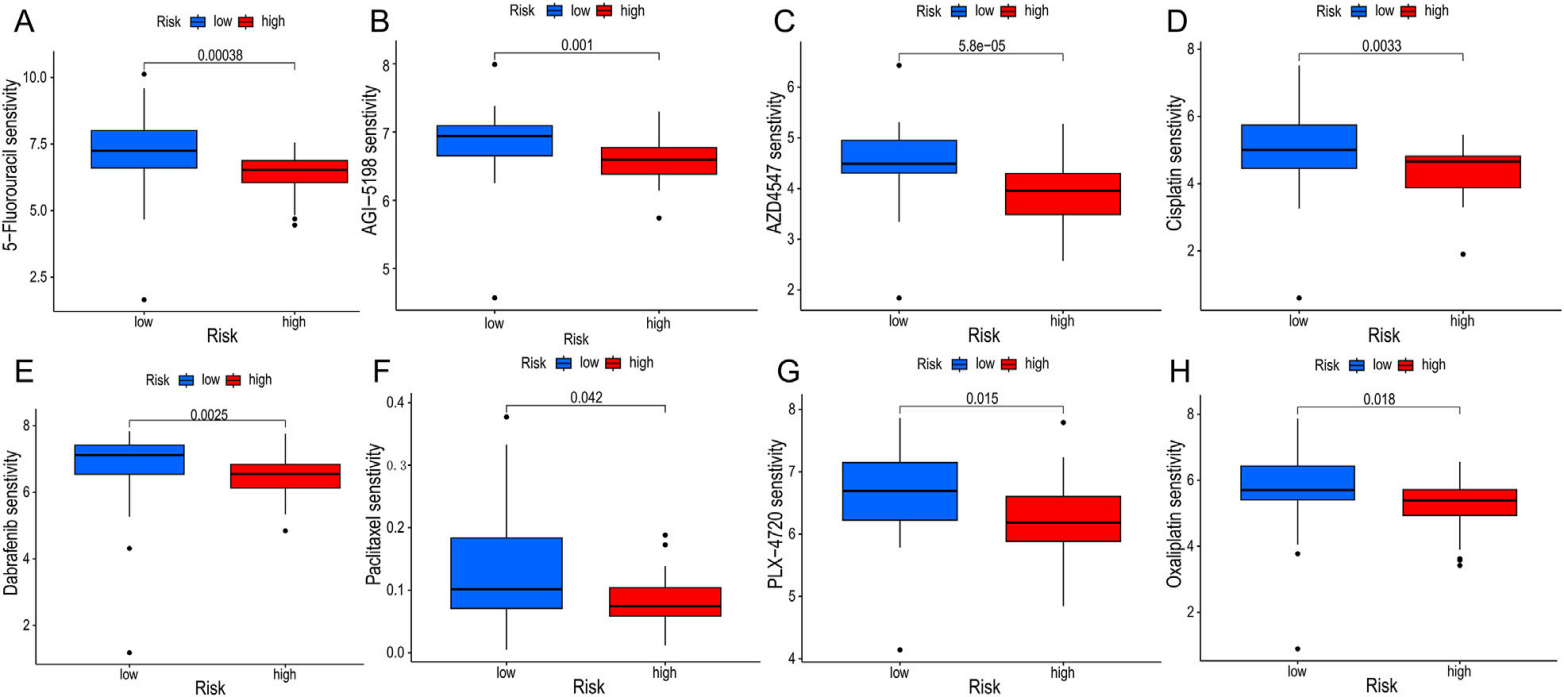
Drug sensitivity analyses. **(A–H)** Differences in half maximal inhibitory concentration (IC50) of commonly used chemotherapeutic drugs and targeted drugs between groups.

We adopted several algorithms including TIMER, EPIC, QUANTISEQ, ESTIMATE and CIBERSORT to comprehensively explore the immune cell infiltration in different PCD-associated Risk Score groups of cholangiocarcinoma patients. Notably, there was a significant positive correlation between PCD-associated Risk Score and anticancer immune-associated cells such as T cells CD8^+^, T cells CD4^+^ Th2 and myeloid dendritic cells (Figure 7A). In contrast, PCDI was negatively correlated with NK cells, activated NK cells and cd4 memory storage cell types (Figure 7A). TME scores showed higher stromalscore, immunescore and estimatescore in the high-risk group than in the low-risk group (Figure 7B). Higher expression of immune checkpoints and HLA-associated genes in the high-risk group suggests a higher likelihood of benefit from immunotherapy (Figures 7C,D). In addition, patients in the high-risk group had lower half-maximal inhibitory concentration values for most commonly used chemotherapeutic and targeted agents for cholangiocarcinoma (e.g., 5-Fluorouracil, AGI-5198, AZD4547, Cisplatin, Dabrafenib, Paclitaxel, PLX-4720, and Oxaliplatin), suggesting that the risk score could be a potential predictor of chemotherapy sensitivity (Figures 8A–H).

## 4 Discussion

The present study comprehensively investigates the role of multiple modes of PCD in CHOL by integrating bioinformatics approaches. The Lasso regression constructed an efficient PCD-related gene signature with stable and robust performance in predicting OS in CHOL patients. Our findings highlighted the significant impact of PCD-associated Risk Score on prognosis, TME and drug sensitivity in CHOL patients.

The construction of the PCD-associated Risk Score using genes like *NCK2*, *BNIP3*, and *BIK* provided a robust prognostic tool, demonstrating its predictive power across different patient cohorts. These genes have been revealed to be associated with cancer development in previous studies, and in particular *BNIP3*, a mitochondrial pro-apoptotic protein in the *Bcl-2* superfamily, has been shown to mediate hypoxia-associated autophagy and thus enhance cancer metastasis (Thongchot et al., 2014; Chourasia and Macleod, 2015). A recent study has shown that BNIP3 is not only associated with autophagy but also reduces MHC-I expression in pancreatic cancer cells to mediate tumor immune escape (Zhou et al., 2025). Furthermore BNIP3 was found in melanoma to drive metabolic reprogramming of cancer cells by enhancing oxidative phosphorylation and inhibiting glycolysis thereby promoting melanoma invasion (Sun et al., 2025). Encouragingly, a Genome-wide association studies (GWAS) and experimental studies also identified BNIP3 as a key gene involved in the process of cholangiocarcinoma-to-sarcoma transdifferentiation (Seol et al., 2011). In view of the above support in the literature, we hypothesize that BNIP3 can also affect tumor aggressiveness in cholangiocarcinoma by influencing the immune microenvironment, mitochondrial hypoxia autophagy, and metabolic reprogramming, among other tumor biological behaviors, thereby affecting tumor aggressiveness and patient prognosis. Moreover, BNIP3 has been identified as a potential biomarker for the prognosis and diagnosis of a wide range of tumors (Yu et al., 2023), which makes our results more convincing. In another study on ferroptosis in cholangiocarcinoma BNIP3 was identified as a key gene involved in ferroptosis and was used to construct a prognostic risk model for cholangiocarcinoma patients (Yao et al., 2022). BIK is a founding member of the BH3 family of pro-apoptotic proteins, and its high expression is usually studied to promote apoptosis (Chinnadurai et al., 2008), but it has been suggested that uncontrolled BIK-mediated chronic low-level cell death may lead to tumor cell adaptation and the evolution of aggressive tumor cells (Pandya et al., 2020). In a study of rectal cancer, BIK was also identified as a poor prognostic marker for microsatellite-stabilized colorectal cancer harboring KRAS mutations, and rectal cancer patients with high expression of BIK tended to have shorter survival times (Liu P. et al., 2022). In the present study BIK was highly expressed in the high-risk patients, and we hypothesized that this might be related to the adaptation of cholangiocarcinoma cells to BIK-mediated apoptosis. NCK2 is a hinge protein containing the -SH2 and -SH3 structural domains, which has been found to promote extracellular matrix degradation and cancer invasion (Chaki et al., 2019). Another study on melanoma indicated that Nck2 promotes tumor cell proliferation, migration and invasion *in vitro* and primary tumor growth *in vivo* (Labelle-Côté et al., 2011). Previous studies on NCK2 in cancer are fewer still need to be further explored. In conclusion, the PCD-associated Risk Scores comprising these three genes accurately predicted the prognosis of CHOL patients, which affirmed their roles in the prognostic stratification of CHOL, but additional experiments are necessary to deeply explore the functions and mechanisms of these genes in CHOL patients.

Functional and pathway enrichment analyses revealed that differentially expressed genes in high-risk patients were predominantly involved in immune-related processes such as cytokine-cytokine receptor interaction and B cell receptor signaling, which are essential for immune modulation within the TME (Zhao et al., 2024). Recent studies have shown that cholangiocarcinoma cells exchange autocrine/paracrine signals with infiltrating cells that populate the microenvironment. This crosstalk is controlled by various cytokine, chemokine and growth factor-mediated signals (Fabris et al., 2021; Ehling and Tacke, 2016). In addition, anti-tumor B lymphocytes can control tumor progression by producing cytokines that promote the formation of tertiary lymphoid structures (TLS) at sites of chronic inflammation, and TLS are considered prognostic markers for predicting longer patient survival (Milardi and Lleo, 2023; Teillaud and Dieu-Nosjean, 2017; Gago da Graç et al., 2021; Reuschenbach et al., 2009).

As terminal cholangiocarcinoma is difficult to remove surgically, immunotherapy has been one of the most promising therapies for patients with end-stage (Greten et al., 2023). Our study found that subgroups at high PCD risk expressed more immune checkpoint genes, suggesting that they are more likely to benefit from immunotherapy (Lin and Yan, 2021). Notably, patients with PCD-associated Risk Score showed greater sensitivity to chemotherapeutic agents such as cisplatin and paclitaxel, as well as to targeted agents such as Dabrafenib, highlighting the potential of PCD-associated Risk Score in guiding personalized therapeutic strategies and providing more precise medication recommendations for CHOL patients.

## 5 Limitation

In this study, a new and efficient prognostic model for cholangiocarcinoma patients was constructed around the PCD gene based on public sequencing data, but this experiment still has some limitations due to the lack of clinical samples. Firstly, due to the lack of clinical samples, the model could not be validated in a large-scale prospective cohort; secondly, the results of this study need to be confirmed in more diverse populations; And lastly, the functions of the three key genes are still unclear, and the mechanism of their roles in cholangiocarcinoma needs to be further investigated by intervening in these genes.

## 6 Conclusion

Although our comprehensive analysis underscores the critical role of PCD-related genes in determining the prognosis, immune landscape, and drug sensitivity of cholangiocarcinoma patients, offers hope for individualised treatment of cholangiocarcinoma patientsthe, results of the study still need to be confirmed using a new cohort and fresh specimens, as all the expression data were obtained from public databases.

## Data Availability

The original contributions presented in the study are included in the article/Supplementary Material, further inquiries can be directed to the corresponding author.

## Supplementary material

SUPPLEMENTARY FIGURE S1
**(A)** Heatmap of the top 50 PCD-related genes with differential expression in cholangiocarcinoma and paracarcinoma tissues. **(B)** Protein-protein interaction diagram of 111 PCD-DEG genes.
