# Supplementary figures and images for: Prognosis of cholangiocarcinoma patients based on multiple patterns of programmed cell death, integrated analysis of the immune microenvironment and drug sensitivity
Source: Front Genet. 2025 May 14;16:1457204. doi: 10.3389/fgene.2025.1457204 (PMC12116470; doi:10.3389/fgene.2025.1457204)

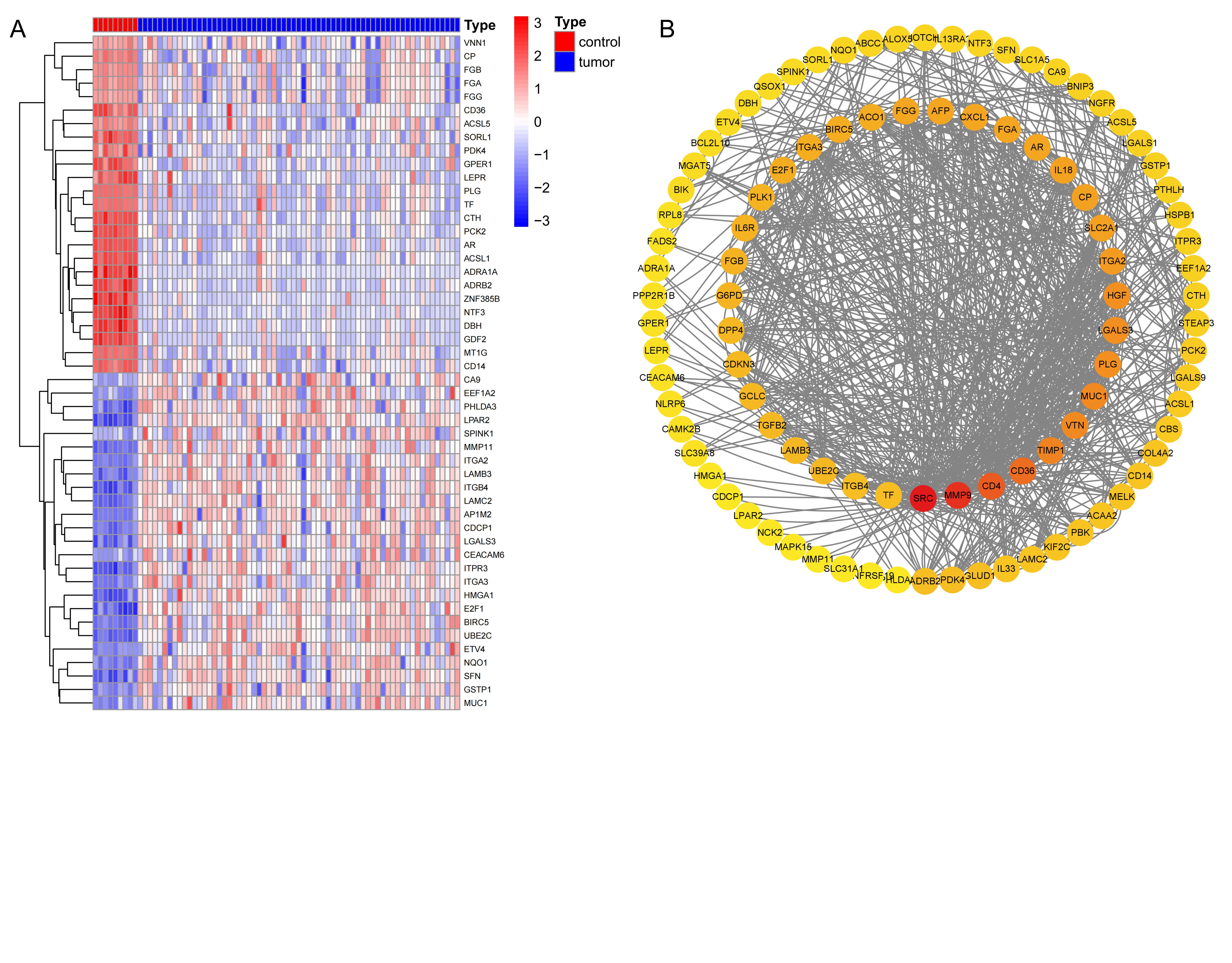

Supplement: Supplementary file 1 [file Image1.jpeg]
